# A dataset of subjective frequency estimates of the words of the peabody vocabulary test by Arabic–English bilingual speakers

**DOI:** 10.1016/j.dib.2024.111056

**Published:** 2024-10-22

**Authors:** Maura Pilotti, Arifi M. Waked, Alaa Mahmoud, Hams Hashim, Dana Alzahid

**Affiliations:** a Department of Sciences and Human Studies, Prince Mohammad Bin Fahd University, P.O. Box 1664, Al Khobar 31952, Saudi Arabia; b Department of Computer Science, Prince Mohammad Bin Fahd University, P.O. Box 1664, Al Khobar 31952, Saudi Arabia; c Cognitive Science Center, Prince Mohammad Bin Fahd University, P.O. Box 1664, Al Khobar 31952, Saudi Arabia

**Keywords:** Word properties, Subjective word frequency, Objective word frequency, Peabody vocabulary test, Bilingual Speakers

## Abstract

A data set was developed for research on word recognition and comprehension as well as for vocabulary knowledge estimation of Arabic–English bilingual speakers (i.e., an understudied population). Subjective frequency (i.e., familiarity) ratings, which reflect language exposure, were collected from 1000 Arabic–English speakers (age range: 18–25) for the printed words of the Peabody Picture Vocabulary Test (PPVT). They were obtained from speakers who had received at least a score of 6 (i.e., competent user of the English language) on the International English Language Testing System (i.e., IELTS). Reading comprehension scores were also obtained from 61 speakers from the same population. In the developed data set, printed words are displayed with their subjective frequency ratings and comprehension scores alongside objective word properties.

Specifications Table

**Table:** 

Subject	Applied Psychology; Education.
Specific subject area	Language processing
Type of data	The data set comprised the 488 printed words of the PPVT-5. For each word, the following information was obtained from Arabic-English bilingual speakers: *mean subjective frequency* ratings (*n* = 1000) and *mean comprehension score* (*n* = 61). Each word was also linked to objective properties gathered from the extant literature [1,2] to illustrate the information upon which such ratings relied.
Data collection	The printed words of the Peabody Picture Vocabulary Test, fifth edition (PPVT-5; [3]) were selected to serve as the stimuli of the data set because of the range of semantic categories and parts of speech (verbs, nouns, and attributes) covered. To collect *subjective frequency ratings*, the 244 words of either form A or B were randomized and assembled into 4 sets of 61 words each (A1, A2, A3, A4, B1, B2, B3, and B4). To avoid repetitions, each participant received a subset from Form A and a subset from Form B to rate (122 words). The assignment of A and B subsets was counterbalanced across participants to minimize order effects. Subsets were posted online for participants to rate. To collect *word comprehension scores,* participants received either Form A or Form B in a counterbalanced order. For each of the 244 words of Form A or B, participants were shown an array of four drawings. One of the drawings corresponded to the meaning of the word, whereas the others were appealing distractors. Drawings were those of the PPVT-5 [3]. On each of the 244 trials, participants were shown a printed word and simultaneously 4 drawings from which they were to select the drawing that best corresponded to the word prompt.
Data source location	The data were collected from Saudi Arabian undergraduate students enrolled at an English-medium institution of higher education. The data were then stored at a secure location at Prince Mohammad bin Fahd University*.*
Data accessibility	The data can be accessed from the Mendeley Repository Repository name: A Dataset of Subjective Frequency Estimates of the Words of the Peabody Vocabulary Test by Arabic-English Bilingual Speakers. Data identification number: 10.17632/hdw92tf943.3 Direct URL to data: https://data.mendeley.com/datasets/hdw92tf943/3
Related research article	

## 1 Value of the Data

• Studies devoted to the word recognition and comprehension processes of Arabic–English bilingual speakers are needed as the linguistic processes of such speakers are largely understudied.
• In speakers for whom English is the first language, objective frequency counts and subjective frequency ratings of printed words are used to predict word recognition and comprehension, including vocabulary knowledge [4]. It is unclear though whether such counts can serve to predict the vocabulary knowledge of bilingual Arabic–English speakers in college.
• The printed words of the PPVT-5 reflect a comprehensive array of semantic categories and key syntactic forms (verb, noun, and attributes). Thus, the subjective frequency ratings of these words can be useful to studies devoted to the word recognition and comprehension processes of Arabic–English bilingual speakers.
• The relationships between subjective frequency ratings and objective word properties can inform researchers about the sources of information upon which such ratings rely. Furthermore, information about the variables that shape word comprehension can be gathered from the relationships that word comprehension scores possess with subjective frequency and objective word properties.
• Appropriately ordered by subjective frequency, PPVT words can also be used to estimate the vocabulary knowledge of Arabic–English bilingual speakers. Vocabulary knowledge is known as an important contributor to language proficiency in general and reading comprehension or writing in particular [[5], [6], [7], [8]]. Thus, information about students’ vocabulary knowledge can help develop effective remedial instruction.

## 2 Background

In research on psycholinguistic processing, subjective and objective frequency counts along with other word properties are often used to select the stimuli to display to participants [9,10]. Systematic selection of such properties within an experimental setting is key to our understanding of word processing phenomena (e.g., the speed with which a word is recognized; [11]). Not surprisingly, English is the language for which most objective and subjective word-level measures have been collected to develop databases of different sizes and properties [10]. For these databases, subjective frequency counts generally reflect the degree of exposure to the first language of the speakers. Yet, research points to consistent individual differences in word frequency counts based on the language exposure of the participants [10]. Thus, it is reasonable to question the extent to which objective and subjective measures reflect the linguistic processing of speakers for whom English is the second language. Of course, for such speakers, different levels of proficiency exist, thereby making the development of datasets of word properties arduous.

Interestingly, the IELTS competent-speaker level is a common basic standard for university admission at English-medium universities around the world. It is also the level that is less studied as much research on word recognition and comprehension of second-language English learners is devoted to novices [7,12]. Most research also entails younger age groups [e.g., 13]. Thus, the vocabulary knowledge and word frequency estimates of speakers at the IELTS competent level can be used as a benchmark to (a) experimentally assess the nature of word recognition and comprehension processes of second-language speakers admitted to undergraduate programs at English-medium institutions; (b) experimentally assess differences in word recognition and comprehension processes of more or less competent speakers from the same population; and (c) fine-tune the nature of instructional remedies for second-language learners at English-medium institutions.

## 3 Data Description

Data are organized in a spreadsheet that contains the 488 words of the PPVT-5 separated into two subsets (Form A and Form B). In the spreadsheet, each word is displayed with the following information: *subjective frequency counts* and *word comprehension scores* by Arabic–English speakers, *objective frequency counts* (i.e., Kucera & Francis norms [14]; HAL norms [15]), *HAL* log *frequency* [15], *length, number of orthographic neighbors, mean* log *HAL frequency of orthographic neighbors, concreteness ratings* [16], *semantic neighborhood density* [17], and *part of speech* (noun, verb, or attribute [3]) (Table 1).

**Table 1:** Each variable's label in the data set along with its description.

Variable's label	Description
Language	English
Form	PPVT5’s Form: A or B
Word	Lexical entry
PPVT5_order	The order in which words are presented in each of the PPVT5’s Forms (A or B)
*Participants’ Responses*	
Subj_Freq	Subjective frequency (i.e., word familiarity)
WC	Word comprehension
*Objective Properties*	
Length	Word length
F_KF	Frequency (Kucera & Francis)
F_HAL	Frequency (HAL; Burgess & Lund, 1997)
Log_F_HAL	Log frequency (HAL; Burgess & Lund, 1997)
ortho_N	No. of orthographic neighbors (i.e., the number of words generated by changing one letter without changing the other letters or their locations)
F_N	Log frequency of orthographic neighbors
Concreteness	Concreteness ratings (i.e., the extent to which the meaning of a word is understood through perception and action; Bysbaert et al., 2014)
SNDensity	Semantic neighborhood density (i.e., the mean distance between a word and its co-occurring semantic neighbors; Shaoul & West, 2010)^a^
PartOfSpeech	Part of Speech (noun, verb, or attribute; Dunn, 2019)

a According to Al Farsi [18], a word that has many close semantic neighbors is more similar to its neighbors with regard to contextual usage (i.e., such words appear quite frequently in similar contexts).

## 4 Experimental Design, Materials and Methods

The printed words of the Peabody Picture Vocabulary Test, fifth edition (PPVT-5; [3]) were selected for their reflecting a variety of semantic categories and key syntactic forms. A convenience sample of 1000 Arabic-English bilingual speakers completed a subjective frequency task. A convenience sample of 61 Arabic–English bilingual speakers who did not partake in the frequency task completed the word comprehension task. Participants were undergraduate students of Saudi Arabian descent who were enrolled in general education courses at an English-medium university. Participants’ ages ranged from 18 to 25 years. To qualify for university enrollment, participants had to demonstrate proficiency in the English language (e.g., writing, reading, speaking, and listening) as per the International English Language Testing System (i.e., IELTS). The minimum overall proficiency score to enroll was 6 (i.e., competent user). According to IELTS, a competent user has “an effective command of the language despite some inaccuracies, inappropriate usage, and misunderstandings”. A competent user also can “use and understand fairly complex language, particularly in familiar situations”. The selection of such speakers was intentional. That is, we wanted to develop a data set for a population of second-language undergraduate students who are commonplace on college campuses of English-medium universities of the Middle East since such a classification is generally required for admission.

The stimuli of the subjective word frequency task were the printed words of the PPVT-5 [3]. They consisted of two equivalent sets of 240 single test words (i.e., Forms A and B) arranged in order of increasing difficulty. Each set was preceded by 4 practice words. Words included verbs, nouns, and attributes covering 20 semantic categories (i.e., actions, adjectives, animals, books and money, body parts, buildings, clothing and accessories, emotions, foods, fruits and vegetables, geographical scenes, household objects, musical instruments, people, plants, shapes and symbols, tools, toys and recreation, vehicles, and workers). Form A had 157 nouns, 53 verbs, and 34 attributes. Form B had 156 nouns, 56 verbs, and 32 attributes.

The 244 words of either form A or B were randomized and assembled into 4 sets of 61 words each (A1, A2, A3, A4, B1, B2, B3, and B4). To avoid repetitions, each participant received an A subset and a B subset to rate (122 words). The assignment of A and B subsets was counterbalanced across participants to minimize order effects. Subsets were posted online for participants to rate. Before accessing sets, the following instructions were administered [19]:

“Words differ in how commonly or frequently they have been encountered. Some words are encountered very frequently, whereas other words are encountered infrequently. The purpose of this study is to rate the frequency of a list of 122 words. We believe that your ratings will be important to future studies involving word recognition. You should base your ratings on the following 7-point scale: 0 = never, 1 = once a year, 2= once a month, 3 = once a week, 4 = every two days, 5 = once a day, and 7 = several times a day”.

The stimuli of the word comprehension task were also the printed words of the PPVT-5. However, participants received either Form A or Form B (i.e., 244 words). The assignment of A and B was counterbalanced across participants. For each word, participants were shown an array of four drawings simultaneously presented. One of the drawings corresponded to the meaning of the word, whereas the others were appealing distractors. Drawings were those used in the PPVT-5 [3]. Participants’ task was to select the drawing that corresponded to the meaning of the target word. Thus, responses were labeled as correct (1) or incorrect (0).

For each stimulus word of either Form A or Form B, objective word properties were gathered from https://elexicon.wustl.edu/about.html [1,2]. The goal was to offer an overview of some of the sources of information upon which familiarity (i.e., subjective frequency) ratings and word comprehension performance relied. Other corpora of objective word properties could be used to link additional variables to the word-level data produced by the Arabic-English speakers selected for the current database.

Table 2 displays descriptive statistics of the database. Table 3, Table 4 illustrate the relationships between variables.

**Table 2:** Mean (M), Standard Deviation (SD), and number of words included in each feature (n).

Descriptive Features	Form A			Form B		
	*M*	*SD*	*n*	*M*	*SD*	*n*
Subjective Frequency Ratings	2.60	0.91	244	2.56	1.10	244
Length	6.84	2.16	244	7.02	2.24	244
Frequency (Kucera & Francis)	23.79	46.72	200	39.25	136.88	201
Frequency (HAL)	8737.11	24,663.13	244	17,338.93	64,352.39	239
Log Frequency (HAL)	7.47	1.97	244	7.49	2.23	239
*No.* Orthographic Neighbors	3.18	4.70	244	3.48	5.50	239
Freq. Orthographic Neighbors	6.31	2.03	145	6.43	2.05	131
Concreteness Ratings	4.31	0.75	196	4.24	0.82	195
Semantic Neighborhood Density	0.53	0.11	221	0.52	0.12	222
Word comprehension	0.66	0.35	244	0.66	0.35	244

**Table 3:** Pearson correlation between mean subjective frequency ratings and descriptive features (*p* < 0.05).

Descriptive features	Form A Fam. Ratings			Form B Fam. Ratings		
	*M*	*CoD*	*n*	*M*	*CoD*	*n*
Length	−0.29	8 %	244	−0.40	16 %	244
Frequency (Kucera & Francis)	+0.51	26 %	200	+0.40	16 %	201
Frequency (HAL)	+0.45	20 %	244	+0.44	19 %	239
Log Frequency (HAL)	+0.71	50 %	244	+0.79	62 %	239
*No.* Orthographic Neighbors	+0.34	12 %	244	+0.39	15 %	239
Freq. Orthographic Neighbors	*ns*		145	*Ns*		131
Concreteness Ratings	*ns*		196	*Ns*		195
Semantic Neighborhood Density	+0.55	30 %	221	+0.62	38 %	222

*Note: n* = number of items included; *CoD* = Coefficient of determination.

**Table 4:** Pearson correlation between mean word comprehension (WC) and descriptive features (*p* < 0.05).

Descriptive features	Form A WC			Form B WC		
	*M*	*CoD*	*n*	*M*	*CoD*	*n*
Subjective Frequency	+0.70	49 %	244	+0.72	52 %	244
Length	−0.44	19 %	244	−0.37	14 %	244
Frequency (Kucera & Francis)	+0.34	12 %	200	+0.21	4 %	201
Frequency (HAL)	+0.28	8 %	244	+0.24	6 %	239
Log Frequency (HAL)	+0.68	46 %	244	+0.67	45 %	239
*No.* Orthographic Neighbors	+0.35	12 %	244	+0.29	8 %	239
Freq. Orthographic Neighbors	+0.22	5 %	145	*ns*		131
Concreteness Ratings	+0.30	9 %	196	+0.28	8 %	195
Semantic Neighborhood Density	+0.51	26 %	221	+0.49	24 %	222

*Note: n* = number of items included; *CoD* = Coefficient of determination.

## Limitations

The subjective frequency ratings were produced by Arabic–English bilingual speakers whose IELTS scores deemed them competent speakers of the English language. Their performance represents an intermediate level of competence and a required one for university admission. Although a reasonable reference point for judging other less or more competent speakers’ vocabulary knowledge and their degree of exposure to English words, such a reference point may be inadequate for speakers of other foreign languages*.*

## Ethics Statement

The data-collection process was approved by the Deanship of Research of the hosting institution to comply with the guidelines of the Office for Human Research Protections of the U.S. Department of Health and Human Services. There is no number or code associated with this ethical approval. PMU Deanship of Research either grants or denies approval (yes/no vote).

Informed Consent was obtained from participants. The informed consent form was deemed to comply with the APA guidelines for the ethical treatment of research participants.

## CRediT authorship contribution statement

**Maura Pilotti:** Conceptualization, Methodology, Data curation, Formal analysis, Writing – review & editing. **Arifi M. Waked:** Conceptualization, Methodology, Data curation, Formal analysis, Writing – review & editing. **Alaa Mahmoud:** Conceptualization, Methodology, Data curation, Formal analysis, Writing – review & editing. **Hams Hashim:** Conceptualization, Methodology, Data curation, Formal analysis, Writing – review & editing. **Dana Alzahid:** Conceptualization, Methodology, Data curation, Formal analysis, Writing – review & editing.

## Data Availability

Mendeley DataA Dataset of Subjective Frequency Estimates of the Words of the Peabody Vocabulary Test by English-Arabic Bilingual Speakers (Original data).
